# Fine mapping of *RBG2*, a quantitative trait locus for resistance to *Burkholderia glumae*, on rice chromosome 1

**DOI:** 10.1007/s11032-015-0192-x

**Published:** 2015-01-20

**Authors:** Ritsuko Mizobuchi, Hiroyuki Sato, Shuichi Fukuoka, Seiya Tsushima, Masahiro Yano

**Affiliations:** 1 National Institute of Agrobiological Sciences, 2-1-2 Kannondai, Tsukuba, Ibaraki 305-8602 Japan; 2 National Agriculture and Food Research Organization, Kyushu Okinawa Agricultural Research Center (NARO/KARC), 496 Izumi, Chikugo, Fukuoka 833-0041 Japan; 3 National Institute of Agro-Environmental Sciences, 3-1-3 Kannondai, Tsukuba, Ibaraki 305-8604 Japan; 4 NARO Institute of Crop Science (NICS), 2-1-18 Kannondai, Tsukuba, Ibaraki 305-8518 Japan

**Keywords:** *Oryza sativa* L., Disease resistance, Bacterial grain rot, QTL, Linkage analysis, Panicle blight

## Abstract

Bacterial grain rot (BGR), caused by the bacterial pathogen *Burkholderia glumae*, is a destructive disease of rice. At anthesis, rice panicles are attacked by the pathogen, and the infection causes unfilled or aborted grains, reducing grain yield and quality. Thus, increasing the level of BGR resistance is an important objective for rice breeding. A quantitative trait locus (QTL) on rice chromosome 1 that controls BGR resistance was previously detected in backcross inbred lines (BILs) derived from a cross between Kele, a resistant traditional lowland cultivar (*indica*) that originated in India, and Hitomebore, a susceptible modern lowland cultivar (*temperate japonica*) from Japan. Further genetic analyses using a BC_3_F_6_ population derived from a cross between a resistant BIL (BC_2_F_5_) and Hitomebore confirmed that a QTL for BGR resistance was located on the long arm of chromosome 1. To define more precisely the chromosomal region underlying this QTL, we identified nine BC_2_F_6_ plants in which recombination occurred near the QTL. Substitution mapping using homozygous recombinant and nonrecombinant plants demonstrated that the QTL, here designated as *Resistance to Burkholderia glumae 2* (*RBG2*), was located in a 502-kb interval defined by simple sequence repeat markers RM1216 and RM11727.

**Electronic supplementary material:**

The online version of this article (doi:10.1007/s11032-015-0192-x) contains supplementary material, which is available to authorized users.

*Burkholderia glumae* causes bacterial grain rot (BGR) and seedling rot in rice (*Oryza sativa* L.), both of which are highly destructive to rice production (Ham et al. 2011). Until now, there have been two reports of quantitative trait loci (QTLs) for BGR resistance (Mizobuchi et al. 2013a; Pinson et al. 2010). As described in Mizobuchi et al. (2013a), we detected a QTL for BGR resistance on the long arm of chromosome 1 by using backcross inbred lines (BILs) derived from a cross between the traditional lowland *indica* cultivar Kele (JP13287) and the modern lowland *temperate japonica* cultivar Hitomebore. The Kele allele at the QTL decreased the ratio of diseased spikelets (RDS).

To validate the effect of the Kele allele at this QTL, we used a resistant BIL (BC_2_F_5_) line (HK19; Fig. 1). Most of the chromosome regions of HK19 originated from the susceptible cultivar Hitomebore, but HK19 also contains a large segment of chromosome 1 and small segments of chromosomes 2, 5, 8, 10, 11, and 12 derived from Kele. Twenty-nine F_2_ plants (BC_3_F_6_) were produced by crossing Hitomebore with HK19. Plants were grown in paddy fields in the summer of 2013 at the National Institute of Agrobiological Sciences (NIAS) in Tsukuba, Japan. Thirty-day-old seedlings were transplanted at a density of one seedling per hill and planted in a single row of 10 hills at a spacing of 18 cm between hills and 30 cm between rows. Basal fertilizer was applied at a rate of 56 kg N, 56 kg P, and 56 kg K ha^−1^. Days from sowing to heading for Kele and Hitomebore, which were transplanted on May 15, were 89 and 94 days, respectively. In contrast, days to heading of the F_2_ plants ranged from 99 to 114 days. Therefore, the F_2_ plants were categorized by heading date and inoculated on different dates (from July 26 to August 7). The Kele and Hitomebore controls were seeded and transplanted on several dates after the F_2_ seeding and transplanting dates to better match the heading dates of the F_2_ plants. We measured resistance to BGR by the modified cut-panicle inoculation method in which panicles containing only spikelets at 1 day after anthesis were harvested and inoculated as previously described (Mizobuchi et al. 2013a). Inoculation and measurement were conducted as previously described (Mizobuchi et al. 2013a). Simple sequence repeat (SSR) markers in the target chromosome regions were screened to identify those detecting polymorphism between Hitomebore and HK19 (IRGSP 2005). The F_2_ plants were then genotyped with 28 SSR markers (Supplemental Table 1). PCR analysis was performed as previously described (Mizobuchi et al. 2013b). Linkage mapping was performed using version 3.0 of MAPMAKER/EXP software (Lander et al. 1987), and the Kosambi map function was used to calculate genetic distances.

**Fig. 1:**
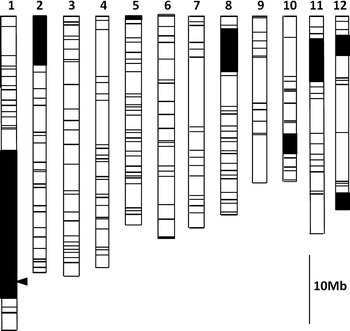
Graphical genotype of a resistant BC_2_F_5_ line (HK19) used for fine mapping of QTLs. Chromosome *numbers* are indicated *above* each linkage map. Positions of marker loci used for genotyping are shown as *horizontal lines* and were obtained from the linkage map of BILs derived from a cross between Kele and Hitomebore (Mizobuchi et al. 2013a). The *arrowhead* next to the long arm of chromosome 1 shows the putative position of the QTL for resistance to bacterial grain rot (BGR) (Mizobuchi et al. 2013a) examined in the present study. *White boxes* indicate regions homozygous for Hitomebore marker alleles; *black boxes* indicate regions homozygous for Kele marker alleles

We performed QTL analyses by using composite interval mapping, as implemented by the Zmapqtl program (model 6) provided in version 2.5 of the QTL Cartographer software (Wang et al. 2005). By QTL analysis, we detected one QTL between RM11725 and RM11727 on the long arm of chromosome 1 (Fig. 2a). The QTL accounted for 35.4 % of the total phenotypic variance in the F_2_ plants, and the Kele allele decreased RDS by 10.4 %. The F_2_ plants derived from the cross of Hitomebore and HK19 showed a wide range of variation (20.6–84.7 %) in RDS (Fig. 2b). The correlation between heading date and RDS was not significant (*R* ^2^ = 0.0562). On the basis of the genotype at RM11727, the SSR marker nearest to LOD peak, F_2_ plants were classified into three genotypic classes; homozygous for the Kele allele, homozygous for the Hitomebore allele, or heterozygous (Fig. 2b). F_2_ plants homozygous for the Kele allele (*n* = 8) showed a low mean RDS (34.8 %), ranging from 20.8 to 66.8 %. Heterozygous plants (*n* = 9) also had a low mean RDS (32.8 %), ranging from 20.6 to 46.4 %. In contrast, the mean RDS was 55.7 %, ranging from 27.9 to 84.7 %, in plants homozygous for the Hitomebore allele (*n* = 12). Thus, plants homozygous for the Kele allele tended to show lower RDS values than those homozygous for the Hitomebore allele. These results clearly support the existence of the previously detected QTL on the long arm of chromosome 1 and show that the Kele allele at the QTL decreases the RDS.

**Fig. 2:**
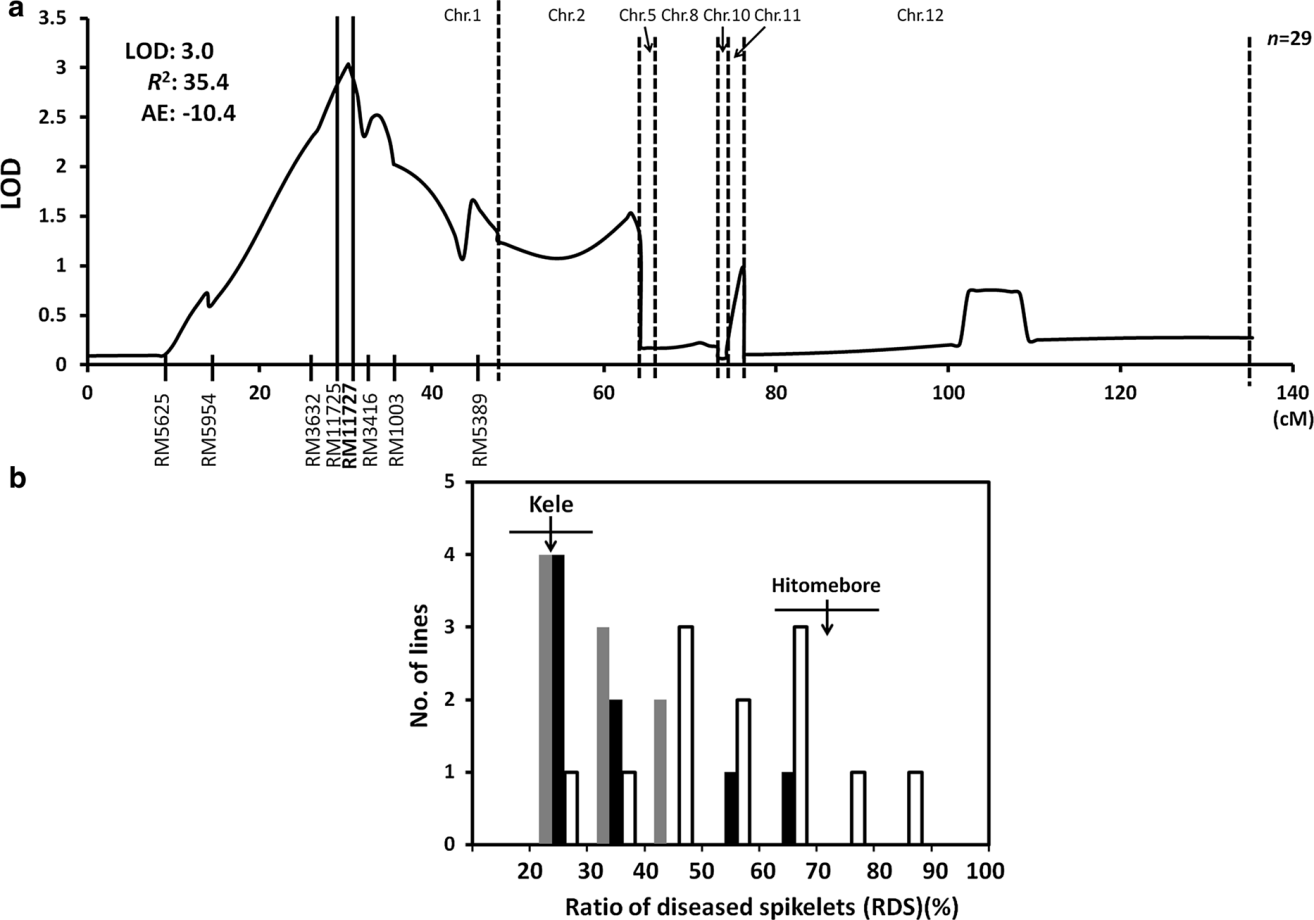
Chromosomal location of a QTL for resistance to bacterial grain rot (BGR) on the long arm of chromosome 1 and effects of allelic differences at linked marker RM11727. **a** The log-likelihood curve indicates a putative QTL position on chromosome 1 in an F_2_ population derived from Hitomebore × HK19 (a resistant BC_2_F_5_ line). We used genome-wide threshold values (*α* = 0.05) to detect putative QTLs on the basis of the results of 1,000 permutations. *LOD* logarithm of odds, *R* ^2^ percentage of phenotypic variance explained, and *AE* additive effect of the allele from Kele relative to that from Hitomebore. **b** Frequency distribution of the ratio of diseased spikelets (RDS) in F_2_ plants showing the three genotype classes of SSR marker RM11727, which was found to be nearest to LOD peak. The *x*-axis labels indicate the maximum RDS in each bin. Genotypes at RM11727 are represented as *white bars* (homozygous for Hitomebore allele), *gray bars* (heterozygous), and *black bars* (homozygous for Kele allele). The RDS values of the F_2_ plants were scored 5 days after inoculation. *Arrows* indicate the mean values for Kele and Hitomebore; *horizontal lines* across the *arrows* indicate the standard deviations

To further delimit the candidate genomic region of the QTL for BGR resistance, we used a BIL (BC_2_F_5_) line (HK114) in which the region of interest on the long arm of chromosome 1 was heterozygous (Supplemental Fig. 1). We identified nine recombinants (BC_2_F_6_) from the BIL line and then selected homozygous recombinant and nonrecombinant plants from the BC_2_F_7_ progeny of each one. Thus, we evaluated nine pairs of lines in the inoculation test. Significant difference about RDS was detected among seven pairs (BC_2_F_7_-12W-1, -2, -3, -5, -6, -7, and -8), whereas two pairs (BC_2_F_7_-12W-4 and -9) had high RDS values that were not significantly different between those of the recombinant and nonrecombinant lines (Fig. 3). Together, the genotype and phenotype information clearly delimit the QTL for BGR resistance between SSR markers RM1216 and RM11727 (a 502-kb interval in the Nipponbare genome reference sequence) on chromosome 1 (Fig. 3). Because we have already identified and named *RBG1* (*Resistance to Burkholderia glumae 1*; formerly named *qRBS1*), a QTL on chromosome 10 involved in resistance to bacterial seedling rot (Mizobuchi et al. 2013b), we have designated this QTL for BGR resistance as *Resistance to Burkholderia glumae 2* (*RBG2*), following the nomenclature recommended by McCouch and CGSNL (Committee on Gene Symbolization 2008).

**Fig. 3:**
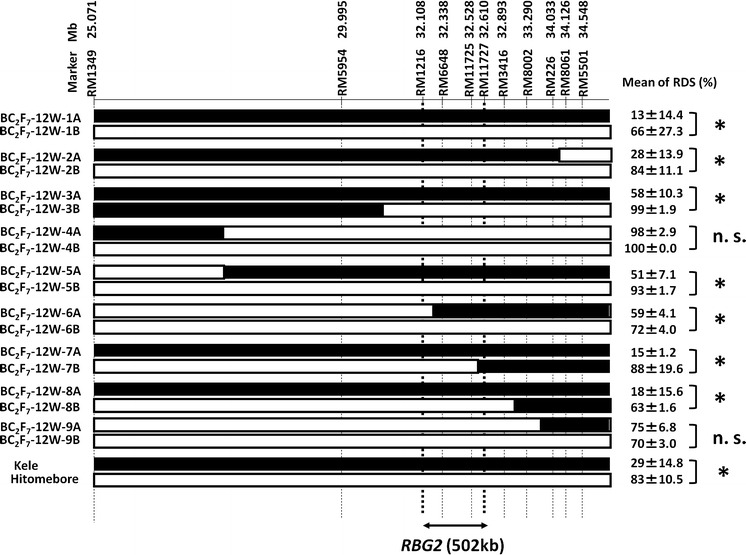
Substitution mapping of a QTL controlling resistance to bacterial grain rot (BGR) on the long arm of chromosome 1 in recombinant BC_2_F_7_ lines. Each pair of lines (e.g., 1A and 1B) was identified from the progeny of a recombinant BC_2_F_6_ plant. *Black* *bars* indicate chromosome regions derived from Kele (resistant); *white* *bars* indicate chromosome regions derived from Hitomebore (susceptible). Positions are based on the International Rice Genome Sequencing Project (IRGSP) 1.0 pseudomolecules of the Nipponbare genome. The location of the candidate QTL (*RBG2*), indicated at the bottom, is based on the phenotypic data obtained in an inoculation test, tabulated on the right. The ratio of diseased spikelets (RDS) scores of the two lines in each pair was compared by using Student’s test. *P < 0.05; *ns* not significant, *P* > 0.05

We surveyed the candidate genomic region of *RBG2* using the Rice Annotation Project Database (http://rapdb.dna.affrc.go.jp/ (Ohyanagi et al. 2006)) to nominate candidate genes. Among the predicted genes, there are none known to be related to disease resistance such as nucleotide-binding-site–leucine-rich repeat (NBS-LRR) genes. It is hard to predict which of the genes might be related to BGR resistance because there have been no reports of genes associated with BGR resistance and because the morphological and physiological functions of *RBG2* are not yet known. Thus, further delimitation of the candidate genomic region of *RBG2* will be necessary to identify the gene corresponding to *RBG2*.

Since *B. glumae* was first discovered in Japan (Goto and Ohata 1956; Goto et al. 1987; Kurita and Tabei 1967; Uematsu et al. 1976), it has also been reported in other countries in East Asia (Chien and Chang 1987; Cottyn et al. 1996a, b; Jeong et al. 2003; Luo et al. 2007; Trung et al. 1993) and Latin America (Nandakumar et al. 2007b; Zeigler and Alvarez 1989). Although several cultivars show partial resistance to BGR (Goto and Watanabe 1975; Groth et al. 2007; Imbe et al. 1986; Mogi and Tsushima 1985; Nandakumar et al. 2007a; Nandakumar and Rush 2008; Pinson et al. 2010; Prabhu and Bedendo 1988; Sayler et al. 2006; Sha et al. 2006; Takita et al. 1988; Wasano and Okuda 1994; Yasunaga et al. 2002), only one report of QTL analysis of BGR resistance has been published other than our previous report (Mizobuchi et al. 2013a; Pinson et al. 2010). This may be because the level of resistance is highly influenced by environmental conditions, making genetic analysis of BGR resistance very difficult (Tsushima 1996; Tsushima et al. 1985). Pinson et al. (2010) found a major QTL on chromosome 3 for BGR resistance colocated with a QTL for heading date. Because late-flowering panicles are subjected to cooler temperatures that are less conductive to disease development during grain fill, it is possible that the genetic effects of the heading-related QTLs affected the disease scoring. On the other hand, by selecting parental cultivars with similar heading dates and using a method (cut-panicle inoculation) that minimizes the effect of heading date variation, we successfully detected a major QTL for BGR resistance on chromosome 1, and no QTL for heading date was detected near this BGR resistance QTL (Mizobuchi et al. 2013a). In the QTL analysis of this study, the correlation between heading date and RDS was not significant (*R* ^2^ = 0.0562). The nine pairs of BC_2_F_7_ lines used for substitution mapping had similar heading dates between homozygous recombinant and nonrecombinant plants. Therefore, we suppose that the disease resistance derived from *RBG2* is not a pleiotropic effect of the QTL for heading date. To enhance our understanding of the genetic control of BGR resistance, we undertook fine mapping of the QTL and successfully defined a candidate genomic region for the QTL, *RBG2*. The *RBG2* map information obtained in this study opens the way not only for the use of *RBG2* in breeding programs, but also for gene isolation that will enable us to elucidate the genetic mechanism of BGR resistance.

## Electronic supplementary material

Below is the link to the electronic supplementary material.

**Supplemental Table 1** Primer pairs used in this study (XLS 32 kb)

**Supplemental Fig. 1** Graphical genotype of a BC_2_F_5_ line (HK114) used for substitution mapping of *RBG2*. Chromosome numbers are indicated *above* each linkage map. Positions of marker loci used for genotyping are shown as *horizontal lines* and were obtained from the linkage map of BILs derived from a cross between Kele and Hitomebore (Mizobuchi et al. 2013a). The *arrowhead* shows RM11727, the nearest marker detected by QTL analysis of an F_2_ population derived from Hitomebore × HK19 (described in Fig. 2). *White boxes* indicate regions homozygous for Hitomebore marker alleles, *black boxes* indicate regions homozygous for Kele marker alleles, and the *gray box* indicates a heterozygous region. (TIFF 76 kb)
