# Supplementary figures and images for: Fine mapping of RBG2, a quantitative trait locus for resistance to Burkholderia glumae, on rice chromosome 1
Source: Mol Breed. 2015 Jan 20;35(1):15. doi: 10.1007/s11032-015-0192-x (PMC4298652; doi:10.1007/s11032-015-0192-x)

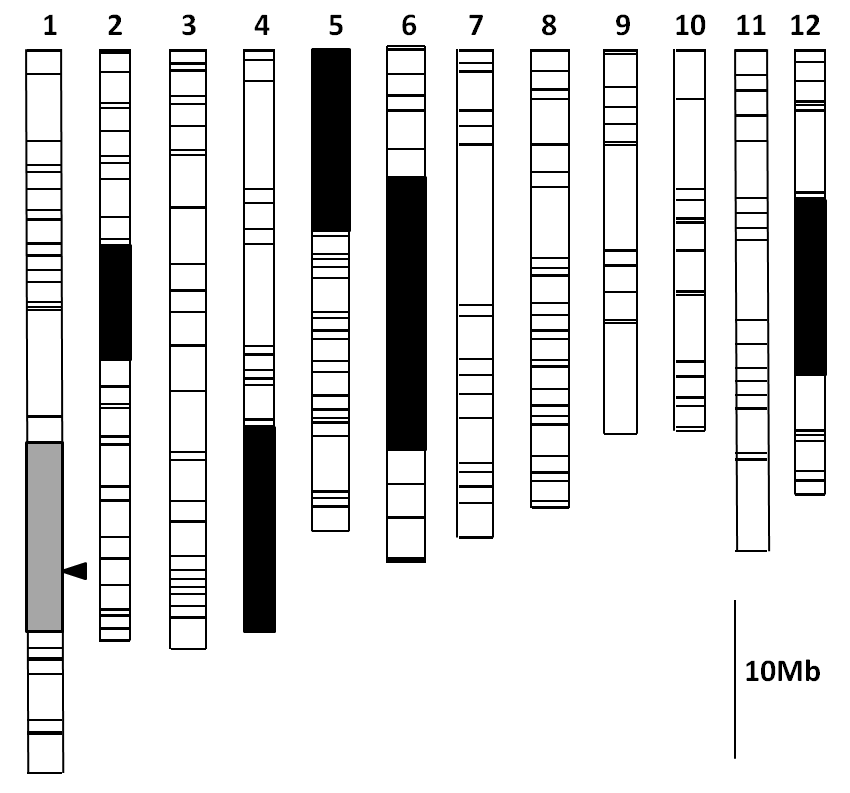

Supplement: Supplementary file 2 — Supplemental Fig. 1 Graphical genotype of a BC2F5 line (HK114) used for substitution mapping of RBG2. Chromosome numbers are indicated above each linkage map. Positions of marker loci used for genotyping are shown as horizontal lines and were obtained from the linkage map of BILs derived from a cross between Kele and Hitomebore (Mizobuchi et al. 2013a). The arrowhead shows RM11727, the nearest marker detected by QTL analysis of an F2 population derived from Hitomebore × HK19 (described in Fig. 2). White boxes indicate regions homozygous for Hitomebore marker alleles, black boxes indicate regions homozygous for Kele marker alleles, and the gray box indicates a heterozygous region. (TIFF 76 kb) [file 11032_2015_192_MOESM2_ESM.tif]
